# Bladder Cancer Immunotherapy: BCG and Beyond

**DOI:** 10.1155/2012/181987

**Published:** 2012-06-20

**Authors:** Eric J. Askeland, Mark R. Newton, Michael A. O'Donnell, Yi Luo

**Affiliations:** Department of Urology, University of Iowa, 375 Newton Road, 3204 MERF, Iowa City, IA 52242, USA

## Abstract

*Mycobacterium bovis* bacillus Calmette-Guérin (BCG) has become the predominant conservative treatment for nonmuscle invasive bladder cancer. Its mechanism of action continues to be defined but has been shown to involve a T helper type 1 (Th1) immunomodulatory response. While BCG treatment is the current standard of care, a significant proportion of patients fails or do not tolerate treatment. Therefore, many efforts have been made to identify other intravesical and immunomodulating therapeutics to use alone or in conjunction with BCG. This paper reviews the progress of basic science and clinical experience with several immunotherapeutic agents including IFN-**α**, IL-2, IL-12, and IL-10.

## 1. Introduction

With more than 73,000 estimated cases diagnosed in 2012, bladder cancer is the fifth most common malignancy in the United States, responsible for more than 14,000 deaths per year [1]. Urothelial carcinoma accounts for 90% of bladder tumors, of which approximately 70% are confined to layers above the muscularis propria—the so-called nonmuscle invasive bladder cancer (NMIBC). These tumors (previously termed “superficial bladder tumors”) include stages Ta, T1, and Tis, occurring in 70%, 20%, and 10% of NMIBC cases, respectively [2]. Standard primary treatment for NMIBC is transurethral resection (TUR); however, recurrence rates for TUR alone can be as high as 70% with up to 30% progressing to muscle invasive disease requiring cystectomy [3]. 

High rates of recurrence and progression have prompted investigation into a myriad of treatments attempting to decrease the burden of this disease. *Mycobacterium bovis* bacillus Calmette-Guérin (BCG) is the most well known and studied of these adjunctive treatments. Since its first description in 1976 by Morales et al. [4], intravesical BCG has become the standard therapy for NMIBC, superior to any other single chemotherapeutic agent for reducing recurrence and preventing progression. Typical complete response rates are 55–65% for papillary tumors and 70–75% for carcinoma *in situ* (CIS), which inversely indicates that 30–45% of patients will be BCG failures [5–7]. Of the complete responders, up to 50% will have a recurrence [8]. Furthermore, side effects range from cystitis and irritative voiding symptoms to much more uncommon life-threatening BCG sepsis. Up to 20% of patients are BCG intolerant due to these side effects [9]. 

**Figure 1:**
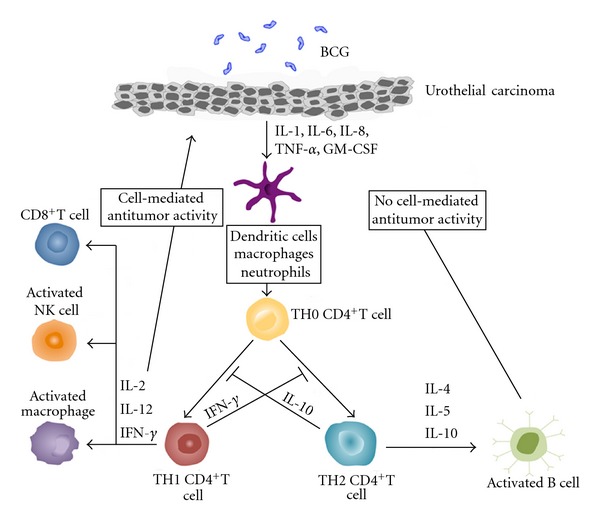
Suggested cascade of immune responses in bladder mucosa induced by intravesical BCG instillation. Attachment of BCG to urothelial cells including carcinoma cells triggers release of cytokines and chemokines from these cells, resulting in recruitment of various types of immune cells into the bladder wall. Activation of phagocytes and the new cytokine environment lead to the differentiation of naïve CD4^+^ T cells into TH1 and/or TH2 cells that direct immune responses toward cellular or humoral immunity, respectively. The therapeutic effect of BCG depends on a proper induction of TH1 immune responses. IL-10 inhibits TH1 immune responses, whereas IFN-*γ* inhibits TH2 immune responses. Blocking IL-10 or inducing IFN-*γ* can lead to a TH1-dominated immunity that is essential for BCG-mediated bladder cancer destruction.

Understanding of BCG, both its mechanisms (which remain incompletely characterized) and its obvious limitations, is critical to improving the efficacy of therapy. The initial step after BCG instillation is binding of BCG to fibronectin expressed on the urothelium, after which the *mycobacterium* is internalized by both normal and malignant cells, resulting in urothelial activation and subsequent inflammatory responses in the bladder [10]. BCG antigens can be presented at the cell surfaces of urothelial and antigen-presenting cells in the context of major histocompatibility complex (MHC) class II, stimulating CD4^+^ T cells and inducing a primarily T helper type (Th) 1 immune response [11]. This complex and robust immune reaction evoked by BCG is evidenced by a massive transient secretion of cytokines in voided urine, including interleukin (IL)-1, IL-2, IL-5, IL-6, IL-8, IL-10, IL-12, IL-15, IL-18, interferon-inducible protein (IP)-10, tumor necrosis factor (TNF)-*α*, granulocyte-monocyte colony stimulating factor (GMCSF), and interferon (IFN)-*γ* [12]. While the role each of these cytokines plays in urothelial carcinoma treatment is not completely clear, Th1 cytokines (e.g., IFN-*γ*, IL-2, and IL-12) have been associated with BCG response, while Th2 cytokines (e.g., IL-10 and IL-6) correlate with BCG failure, as illustrated in Figure 1 [13–16]. Since the advent of BCG therapy, a significant amount of data has accumulated to support maintenance treatments, which typically consist of a series of shorter treatments at 3–6-month intervals, often based on the time table developed by the Southwest Oncology Group [17]. 

While success has improved with the addition of maintenance treatments, the combination of intravesical therapy, surveillance, and repeat surgical procedures place enormous costs on the US healthcare system, approaching $4 billion annually [3]. Prompted by the burden of patients either with BCG refractory disease or who are intolerant of treatment, the search goes on for therapeutic improvements. Given that the effect of BCG is immune mediated, decades of research have focused on adjunctive immunotherapies including IFN-*α*, IL-2, IL-10, and IL-12. This paper summarizes and integrates key points for the clinical urologic oncologist. 

## 2. Interferon-*α*2b

Interferons (IFNs) are glycoproteins initially isolated in the 1950s and valued for their antiviral properties. Three types have been isolated, IFN-*α* (which is actually a family of interferons), IFN-*β*, and IFN-*γ*. IFN-*α* and IFN-*β* are grouped as “Type I” interferons, whereas IFN-*γ* is a “Type II” interferon. The Type I interferon receptor has 2 components, IFNAR-1 and IFNAR-2, which subsequently bind and phosphorylate Jak molecules initiating a cascade resulting in gene transcription [18]. The IFN-*α* family is well known to stimulate natural killer (NK) cells, induce MHC class I response, and increase antibody recognition [19]. They have antineoplastic properties by direct antiproliferative effects and complex immunomodulatory effects [18], both of which could be advantageous for bladder cancer treatment. Clinically available preparations include IFN-*α*2a (Roferon-A, Roche Laboratories, Nutley, NJ) and IFN-*α*2b (Intron-A, Schering Plough, Kenilworth, NJ), though to date most research involves IFN-*α*2b. There has been interest in IFN-*α*2b both alone and in combination with BCG, where a synergistic response has been described. Conceptually, combining BCG and IFN makes sense. BCG efficacy depends on the induction of a robust Th1 cytokine profile, and IFN-*α*2b has been shown to potentiate the Th1 immune response [12]. However, despite theoretical promise, data after translation to clinical practice has been mixed. 

For many years, IFN-*α* was thought to exert antitumor activity primarily through direct antiproliferative properties [20]. At least part of this effect has been shown to be mediated by directly inducing tumor cell death. IFN-*α* has been documented to independently induce Tumor-Necrosis-Factor-Related Apoptosis-Inducing Ligand (TRAIL) expression in UM-UC-12 bladder cancer cells [21], which subsequently triggers apoptosis in cells expressing the appropriate cell death receptor. Cell death occurs ultimately by Fas-associated protein with death-domain- (FADD-) dependent activation of the death inducing signaling complex (DISC) followed by activation of caspase-8. Furthermore, Tecchio and colleagues have demonstrated that IFN-*α* can stimulate TRAIL mRNA as well as the release of a bioactive soluble TRAIL protein from neutrophils and monocytes, which induces apoptotic activity on TRAIL-sensitive leukemic cell lines [22]. It also appears that IFN-*α* apoptotic effects may not be limited to TRAIL; rather it may trigger caspase-8 via both cell death receptor-dependent and independent pathways [23]. Much like IFN-*α*, BCG has also been shown to induce TRAIL [24], which has correlated with patient response to BCG therapy and has been a source of overlapping research interest. Other direct IFN-*α* effects include enhancing cytotoxicity of CD4^+^ T cells, increasing antigen detection by upregulating MHC class I expression [20, 25, 26]. Direct suppression of proliferation by induction of tumor suppressor genes or inhibition of tumor oncogenes has also been described [20]. Also contributing to antiproliferative properties, IFN-*α* has been documented to decrease angiogenesis and basic fibroblast growth factor. Additionally, it downregulates matrix metalloprotease-9 (MMP-9) mRNA as well as the MMP-9 translational protein in murine bladder tumors [27]. Interestingly, it has also been demonstrated that an optimal biologic dose with higher frequency, rather than maximal tolerated dose, produced the most significant decreases in angiogenesis. Significantly decreased angiogenesis has also been documented in human urothelium during and after IFN-*α*2b treatment following transurethral resection of superficial bladder tumors [28].

*In vivo* monotherapy with IFN-*α*2b for bladder cancer in humans has been explored by multiple groups. In 1990, Glashan published data from a randomized controlled trial evaluating high dose (100 million unit) and low dose (10 million unit) IFN-*α*2b regimens in patients with CIS [29]. Patients were treated weekly for 12 weeks and monthly thereafter for 1 year. The high and low dose groups had complete response rates of 43% and 5%, respectively. Of the high dose patients achieving a complete response, 90% remained disease-free at a notably short 6 months of follow-up. The primary side effects of treatment were flu-like symptoms (8% low dose, 17% high dose) but without the irritative symptoms seen so often in BCG therapy. When IFN-*α*2b was investigated alone to treat BCG failures, eight of twelve patients had recurrence at initial three-month evaluation and only one of twelve was disease-free at 24 months [30]. Another trial conducted by Portillo and colleagues randomized 90 pT1 bladder cancer patients to either intravesical treatment or placebo groups as primary prophylaxis after complete resection [31]. They utilized a similar dosing schedule but used 60 million units IFN-*α*2b. At 12 months of followup, recurrence rates were significantly lower for IFN-*α*2b group than placebo, 28.2% versus 35.8%, respectively. However, after 43 months, rates were similar—53.8% and 51.2%, respectively, indicating that treatment benefit of IFN-*α*2b alone may not be durable. 

Given the described antiproliferative and immunomodulatory effects of IFN-*α*, combination therapy with BCG has held tantalizing promise. Gan et al. found significantly greater antitumor activity with combination therapy than BCG alone: 14/15 mice receiving BCG/IFN-*α* versus 8/15 mice receiving only BCG became tumor-free after 5 weekly intralesional treatments [32]. In an **in * vitro* study comparing BCG plus IFN-*α* to BCG alone, our group demonstrated a 66-fold increase in IFN-*γ* production in peripheral blood mononuclear cell (PBMC) cultures [12]. Since IFN-*γ* is a major Th1-restricted cytokine found in patients responding to BCG therapy, it has been used routinely as a surrogate marker for Th1 immune response in studies examining effect of IFN-*α* [12]. It appears that IFN-*α* by itself generates a negligible Th1 response, as no significant levels of IFN-*γ* were detected after IFN-*α* was incubated alone with the PBMCs. We have also demonstrated that the augmented IFN-*γ* production persisted even with reduced doses of BCG. These findings give credence to the idea that adding Th1-stimulating cytokines may allow for a decrease in BCG doses, thereby decreasing side effects thought to be directly related to BCG. Further augmenting Th1 differentiation, IFN-*α* was found to increase levels of several Th1 cytokines, including IL-12 and TNF-*α* as well as decreasing known Th1 inhibitory cytokines IL-10 and IL-6 by 80–90% and 20–30%, respectively [33].

Clinical investigations with the combination of IFN-*α*2b and BCG began initially in BCG refractory patients but were subsequently expanded to BCG naïve patients. Stricker et al. found the combination to be safe, with a similar side effect profile to BCG alone [34]. In 2001, O'Donnell and colleagues reported on combination therapy administered to 40 patients who had failed at least 1 course of BCG alone [35]. At 24 months, 53% of patients were disease-free. Patients with two or more prior BCG failures faired similarly to patients with only one. Lam et al. in 2003 reported on the treatment of 32 patients, of which 20 (63%) were BCG failures. At 22 months' median followup, 12 of the 20 BCG failure patients (60%) remained disease-free [36]. In a smaller trial, Punnen et al. documented a 50% disease-free rate after combination therapy at 12 months' followup in 12 patients with BCG refractory disease [37]. A subsequent large community-based phase II clinical trial examined 1106 patients from 125 sites with NMIBC, which were split into BCG naïve and BCG refractory groups [38]. At median 24 months' followup, tumor-free rates were 59% and 45%, respectively. In this larger trial, patients who had two or more courses of prior BCG therapy had a worse outcome when compared to patients who had 1 or less, likely indicating more resistant disease. A recent study limited to BCG naïve patients demonstrated similar disease-free rate of 62% but with much longer median followup of 55.8 months [39]. Furthermore, after evaluating failure patterns and response rates to BCG plus IFN-*α*, Gallagher et al. found that patients who recurred more than 12 months after initial BCG treatments had similar tumor-free rates at 24 months when compared to BCG naïve patients [40]. However, patients who recurred within a year of receiving their initial BCG treatments did significantly worse, with disease-free rates of 34–43% at 24 months, indicating that additional immunotherapy may not be appropriate. Overall, while promising, these data are unable to define any treatment benefit of combination therapy over BCG alone.

To date, the only randomized trial comparing BCG alone to BCG plus IFN-*α* was a multicenter study of 670 BCG naïve patients with CIS, Ta, or T1 urothelial carcinoma [41]. This was a four-arm trial evaluating efficacy of megadose vitamins as well as BCG and IFN. Patients were randomized to 1 of 4 groups: BCG plus recommended daily vitamins, BCG plus megadose daily vitamins, BCG plus IFN-*α*2b plus recommended daily vitamins, and BCG plus IFN-*α*2b plus megadose daily vitamins. At 24-month followup, median recurrence-free survival was similar across all groups, though the two IFN-*α*2b groups experienced higher incidence of constitutional symptoms and fever (*P* < 0.05).

Lastly, there are multiple areas where additional research is warranted. A recent evolution in combination therapy has been the development of an IFN-*α*2b expressing strain of recombinant BCG (rBCG-IFN-*α*) from the Pasteur strain of BCG. An initial **in * vitro* study documented enhanced IFN-*γ* expression in PBMCs after incubation with rBCG-IFN-*α* as compared to standard BCG [42]. A subsequent study reported that rBCG-IFN-*α* increased cytotoxicity up to 2-fold over standard BCG in PBMC cultures. Both CD56^+^CD8^−^ NK cells and CD8^+^T cells were identified as primary contributors to the increased cytotoxicity [43]. Combining IFN-*α*2b with other antiproliferative agents has shown **in * vitro* promise. Louie et al. reported that a combination of IFN-*α*2b and maitake mushroom D-fraction (PDF) could reduce T24 bladder cancer cell proliferation by 75%, accompanied by G_1_ cell cycle arrest [44]. Another combination recently published this year documented that adding grape seed proanthocyanin significantly enhanced antiproliferative effects of IFN-*α*2b, with >95% growth reduction in T24 bladder cancer cells. Cell cycle analysis also revealed G_1_ cell cycle arrest, with Western blots confirming expression of G_1_ cell cycle regulators [45]. Lastly, several groups have investigated gene therapy with a recombinant adenovirus delivery system (rAd-IFN/Syn3), which could potentially result in sustained therapeutic IFN-*α*2b levels for long periods of time. Nagabhushan et al. were able to demonstrate delivery and expression of IFN in the bladder as well as significant tumor regression in mice. Phase I trials with rAd-IFN/Syn3 were ongoing at the time of their publication in 2007 [46]. 

## 3. Interleukin-2

The discovery and characterization of interleukin-2 (IL-2) was one of the most important breakthroughs in the field of immunology. Prior to its discovery, lymphocytes were thought to be terminally differentiated and incapable of proliferation [47, 48]. In 1975, it was discovered that the supernatant of murine splenic cell cultures could stimulate thymocytes, suggesting a native effector protein was responsible for this mitogenic activity [48, 49]. When initially examined independently by different investigators, this “effector protein” was given multiple working names including thymocyte-stimulating factor (TSF), thymocyte mitogenic factor (TMF), T cell growth factor (TCGF), costimulator, killer cell helper factor (KHF), and secondary cytotoxic T-cell-inducing factor (SCIF) [50]. In 1979, it was recognized that these factors likely represented the same entity, and the nomenclature was standardized with the term “interleukin” (between leukocytes). Thus, the “effector protein” was named IL-2, differentiating it from the only other interleukin known at that time, IL-1 [50]. Regardless of the nomenclature, this protein was recognized to promote proliferation of primary T cells **in * vitro*, which revolutionized the experimental armamentarium in the field of immunology [47, 49, 51].

Since the discovery of IL-2-mediated control of T-cell growth in culture, there has been much progress in elucidating its mechanisms. It was discovered relatively early that IL-2 enhances the production of cytotoxic lymphocytes which are capable of lysing tumor cells while leaving normal cells unharmed [51–53]. These IL-2 activated lymphocytes became known as “lymphokine-activated killer” (LAK) cells and were thought to play a large role in antitumor immune function [51–53]. Additionally, it was noted that IL-2 functions to augment the cytotoxic activity of NK cells and monocytes [54, 55]. It has even been discovered that IL-2 is important for the activation of B cells [56]. As the CD4^+^ Th1 and Th2 cell cytokine profiles were defined, it became clear that IL-2 is predominantly a Th1-secreted cytokine [57].

The cytotoxic antitumor capabilities induced in lymphocytes by IL-2 make it a potential cancer immunotherapeutic agent. To date, multiple studies have demonstrated regression of metastatic disease following systemic IL-2 treatment in some cancers [58]. Rosenberg et al. reported on 157 patients with a heterogenous mix of metastatic cancers refractory to other treatments including renal cell, colon cancer, breast cancer, and lymphoma. Patients were treated with either IL-2 and LAK cells or IL-2 alone. Between the two groups, 9 complete and 20 partial responses were obtained. Significant morbidity has been reported with systemic IL-2 much of which is secondary to increased capillary permeability [58, 59] and includes weight gain, hypotension, oliguria, elevated creatinine, and bilirubin. These tend to resolve with cessation of IL-2 therapy [58]; however, Rosenberg reported 4 treatment-related deaths among their 157 patients. Despite the reports of morbidity, IL-2 seemed to offer hope to patients with few treatment options.

With regard to bladder cancer, interest was stimulated after multiple investigators identified elevated IL-2 levels (as well as other cytokines) in urine of patients following BCG, suggesting an immunomodulatory effect of BCG [60–67]. Additionally, an elevation in IL-2 receptor expression has been documented on T cells in voided urine after BCG therapy [64, 66]. Increased levels of urinary IL-2 have also been found to correlate with BCG response, which supports the concept that a Th1 cytokine profile confers a favorable response to BCG [15]. Furthermore, elevated IL-2 has been reported in the serum of patients following BCG instillation, which suggests both a local and systemic immune response to therapy [68, 69]. These findings led to the conclusion that IL-2 may have a therapeutic use in bladder cancer.

One of the first clinical trials reported evidence of bladder tumor regression following intralesional injections of IL-2, with no adverse events recorded [70]. Multiple murine studies have demonstrated that systemic administration of IL-2, with or without BCG, can significantly decrease tumor size, suppress tumor growth, and improve mean survival [71–73]. A small clinical study investigating systemic IL-2 administration effects on low-stage bladder cancer found a complete and partial response rate in 5 of 12 patients, though 2 patients discontinued therapy due to toxicity [74]. The poor side effect profile of systemic IL-2 administration subsequently prompted a shift to utilize IL-2 as an intravesical therapy. Reports of intravesical use revealed a much improved side effect profile as well as some efficacy alone or when combined with BCG [75–78]. Den Otter et al. administered intravesical IL-2 alone after incomplete transurethral resection of grade 1-2, T1 papillary urothelial carcinoma, and documented “marker lesion” regression in 8 of 10 patients [79]. Additional experiments have focused on developing recombinant-IL-2-secreting strains of BCG [80–85]. Animal models using this approach have shown that compared to native BCG, IL-2-secreting BCG strains have increased IFN-*γ* production, induced a more favorable IFN-*γ* to IL-4 ratio, improved antigen-specific proliferation, enhanced antitumor cytotoxicity, and mounted a Th1 cytokine profile even in immunosuppressed or IL-4 transgenic mice (two conditions which favor a Th2 response) [80–83, 85]. More recent animal and *in vitro* studies have investigated IL-2 transfecting dendritic cells (DCs), immobilized streptavidin-tagged bioactive IL-2 on the biotinylated surface of murine bladder mucosa, and development of a murine IL-2 surface modified bladder cancer vaccine [86–89]. Since IL-2 plays a crucial role in the Th1 response, it will continue to be a source of interest for immunotherapy of bladder cancer.

## 4. Interleukin-12

Interleukin-12 (IL-12) has been the focus of significant cancer research among cytokines as well. In 1987, it was discovered through **in * vitro* experiments that there existed a factor which synergized with IL-2 in promoting a cytotoxic T lymphocyte (CTL) response [89]. This factor was given the name cytotoxic lymphocyte maturation factor (CLMF) [89]. Shortly thereafter a factor was discovered that induced IFN-*γ* production, enhanced T cell responses to mitogens, and augmented NK cell cytotoxicity [90]. This factor was provisionally called natural killer cell stimulatory factor (NKSF) [90]. It did not take long to discover that these factors represented the same entity, thus the nomenclature converged and this protein was termed IL-12 [91–95]. 

Although initially discovered in a B cell lymphoma, it was subsequently found that IL-12 is primarily involved with the regulation of T cells, causing proliferation of both activated CD4^+^ and CD8^+^ T cell subsets while causing minimal proliferation of resting PBMCs [90, 92]. This concept is supported by studies demonstrating that the IL-12 receptor is upregulated in activated T and NK cells, but not in activated B cells [95]. IL-12 potentiates a Th1-specific immune response, and it was later discovered that DCs produce IL-12 and thus direct the development of Th1 cells from naïve CD4^+^ T cells [96, 97]. Additionally, IL-12 can, by itself, stimulate the activation of nonspecific LAK cells and facilitate the generation of an allogeneic CTL response [98]. IL-12 has even been found to play a role in the activation of neutrophils [99, 100]. Multiple studies have demonstrated that IL-12 strongly inhibits neovascularization, thought to be mediated through its induction of IFN-*γ* [101–104]. Furthermore, the mechanism by which IL-12 enhances the cytolytic effect of NK cells is primarily via the perforin pathway [105, 106].

Multiple animal studies have shown tumor responsiveness to immunomodulation with IL-12. Using systemic or peritumoral injections, IL-12 showed antitumor properties in murine sarcoma, melanoma, renal cell carcinoma, lung cancer, colon cancer, breast cancer, and bladder cancer models [102, 107–111]. Increases in serum IFN-*γ* were observed in mice treated with IL-12 [108]. Antitumor efficacy was lost in CD8^+^-depleted mice, but not CD4^+^-depleted mice or NK-deficient mice, suggesting that the primary mediators of the antitumor IL-12 effect are CD8^+^ T cells [107, 108]. Some of these studies saw effectiveness even with metastatic disease, including bladder cancer [107, 108, 111]. Multiple murine studies have also revealed added effectiveness with IL-12 administered in combination with chemotherapeutic agents [109, 112–114]. Additionally, IL-12 therapy has shown synergistic activity when combined with radiation therapy in mice [110, 115]. Various delivery systems for IL-12 therapy have been tested in mice using viral and retroviral vectors to elicit an IL-12 response [116–120]. These constructs have shown some effectiveness as anticancer therapeutics [116–119]. IL-12 as an intravesical therapy for bladder cancer has shown great success in mouse models. BCG was found to be a potent stimulus for IL-12 expression, and neutralization of IL-12 significantly dampened the induction of IFN-*γ* by BCG [121]. BCG therapy for murine bladder cancer was essentially found to be ineffective in IL-12 knock-out mice, suggesting a crucial role for IL-12 in the BCG response [122]. When IL-12 is used as a therapy with BCG, it causes a synergistic induction of IFN-*γ* [121]. Intravesical IL-12 treatment alone was found to be effective for the treatment of orthotopically placed bladder tumors in mice, and urinary IFN-*γ* was subsequently found to be significantly elevated [111, 123]. These observations further support the importance of IFN-*γ* induction for effective immunotherapy of bladder cancer. More recently, multiple attempts have been made to improve the delivery of intravesical IL-12 to the bladder mucosa to improve efficacy. One method utilized cationic liposome-mediated IL-12 gene therapy, which showed improved survival and tumor-specific immunologic memory in mice [124]. Another method utilized chitosan, a mucoadhesive biopolymer, to increase IL-12 delivery to urothelial surfaces [125]. This method showed improved efficacy over IL-12 alone in a mouse model [125].

The great success of IL-12 in treating various murine cancers subsequently led to experiments testing its use on human cancers, though with mixed success. Initial trials focused on systemic IL-12 treatment for metastatic cancer, though progress was initially halted when several patients suffered severe toxic effects from the treatment and two patients died from the therapy [126]. A phase I trial of systemically administered IL-12 in 40 patients with advanced malignancy found a dose-dependent increase in circulating IFN-*γ* with administration [127]. Experiments on the peripheral blood of these patients showed augmented NK cell cytolytic activity and enhanced T cell proliferation [128]. Unfortunately, of these 40 patients there was only one partial response and one transient complete response [127]. Further studies looking at chronic administration of twice weekly IL-12 in patients with metastatic cancer found that it is well tolerated and induces costimulatory cytokines (including IFN-*γ*) [129]. However, in a cohort of 28 patients, there was only one patient with a partial response and two with prolonged disease stabilization, with one of these patients eventually exhibiting tumor regression [129]. Similar low response rates have been seen with systemic IL-12 in other studies of advanced malignancies [130–134]. Various combinations of immunotherapy have been tested with systemic IL-12 in humans. A phase I study examined systemic IL-12 with low dose IL-2 and showed it was well tolerated, and the addition of IL-2 significantly augmented IFN-*γ* production as well as the NK response [135]. Of 28 patients, there was one partial response and two pathologic responses [135]. Another phase I study using systemic IL-12 with IFN-*α*2b showed acceptable toxicity, but with no response in 41 patients [136]. As discussed previously, intravesical IL-12 showed great promise for the topical treatment of bladder cancer in a mouse model; however, this success did not translate clinically in humans. A phase I study of intravesical IL-12 therapy in patients with superficial bladder cancer showed minimal toxicity, but disappointing efficacy [137]. A total of 15 patients were enrolled in this study, of which 12 had no visible pretreatment lesions [137]. Of these 12 patients, 7 remained disease-free and 5 had recurrence within 4 weeks. The remaining 3 patients with pretreatment lesions had persistent disease at followup [137]. Perhaps the most disparaging results were that there was negligible IFN-*γ*-induced in the urine and serum of these patients post-treatment, suggesting minimal immunologic effect from intravesical IL-12 therapy [137]. Despite the disappointing results from human studies, IL-12 remains an important target for the treatment of bladder cancer. 

## 5. Interleukin-10

Unlike other cytokines previously discussed, interleukin-10 (IL-10) is distinct in that its primary effect is to promote a Th2 response and thus dampen the immunotherapeutic effects of BCG for the treatment of bladder cancer [54, 138, 139]. As a result, it may have therapeutic value not by its native function, but by abrogation of its native function. IL-10 was first characterized in 1989. It was initially termed cytokine synthesis inhibitory factor (CSIF), a rather fitting name, because it was found to inhibit the production of several cytokines produced by Th1 clones [140]. The most important of these cytokines was IFN-*γ*, which was recognized as an important player in the Th1 response. As discussed previously, it is a key contributor in the immunotherapeutic effectiveness of BCG [140, 141]. Further studies showed that IL-10 prevented a delayed-type hypersensitivity (DTH) response to BCG and the neutralization or abrogation of IL-10 prolonged a response, thus further supporting its role in the Th1/2 response [138, 142]. Several human tumors, including melanoma, non-small-cell lung carcinoma, renal cell carcinoma, and bladder cancer, have been found to express elevated levels of IL-10 [143–147]. It is speculated that production of IL-10 by tumor cells may represent an “escape mechanism” whereby tumor cells avoid Th1-immune-mediated tumoricidal effects [143]. 

There has been significant progress in determining the regulation and mechanism of IL-10 function since its discovery, particularly with regard to its role in tumor immunology. It is secreted by multiple cell types including Th2 cells, B cells, and monocytes/macrophages [140, 148–150]. Like many other cytokines, IL-10 is known to autoregulate itself by downregulating its own mRNA synthesis [150]. It has been shown to block the accumulation of macrophages and DCs at tumor sites, which are important players in the cellular immune response [151, 152]. Additionally, it compromises DCs ability to stimulate T-cells causing induction of antigen-specific anergy of T cells [153]. Furthermore, it down-regulates the expression of MHC class II and costimulatory molecules, thus preventing a cellular immune response to tumor cells [154–156]. During activation of CD4^+^ T cells, the presence of IL-10 can cause them to differentiate into T regulatory cells 1 (Tr1), leading to peripheral tolerance [157]. IL-10 further reduces cellular tumoricidal activity by preventing release of reactive nitrogen/oxygen intermediates by macrophages and NK cells, a key step in their efficacy during cellular immune defense [139, 158]. 

Successful treatment of bladder cancer with BCG, as discussed previously, requires a Th1 cytokine profile. IL-10 antagonizes the production of a Th1 milieu, thus its neutralization has been targeted as a potential means to augment the BCG response. Several murine studies have demonstrated that after IL-10 knockout mice are inoculated with bladder cancer, they have an improved BCG and local immune response, increased bladder mononuclear infiltrate, enhanced DTH responses, greater antitumor activity, and prolonged survival [54, 138, 143]. Although murine IL-10 knockout studies are conceptually important, studies focused on IL-10 neutralization hold more promise as clinically useful therapeutics. Murine bladder cancer studies utilizing anti-IL-10 neutralizing antibody have shown similar results, with BCG treatment inducing an enhanced DTH response and increased bladder mononuclear infiltrate [138, 142]. More recent efforts have been placed at targeting the IL-10 receptor. The IL-10 receptor is composed of two known subunits (IL-10R1 and IL-10R2), and the IL-10R1 subunit plays the predominant role in signal transduction [159]. In *in vitro* studies, we have recently shown that splenocytes incubated with BCG and anti-IL-10-receptor 1 monoclonal antibody (anti-IL-10R1 mAb) produced significantly higher IFN-*γ* than those incubated with BCG plus anti-IL-10-neutralizing antibody, suggesting that interference with IL-10 signal transduction may be more effective than neutralizing IL-10 protein (17). In *in vivo* studies, mice treated with BCG and anti-IL-10R1 mAb showed increased urinary IFN-*γ* production compared to BCG controls [160]. In a similar murine experiment, there was improved overall and tumor-free state in mice treated with BCG plus anti-IL-10R1 mAb compared to BCG treatment controls, though this difference did not reach statistical significance [160]. Most recently, in an experiment designed to follow murine survival after inoculation with a luciferase-expressing MB49 bladder cancer cells, we discovered that control mice and BCG-only treated mice developed histologically confirmed lung metastasis, whereas mice treated with BCG and anti-IL-10R1 mAb developed no metastasis [unpublished data]. This difference was statistically significant and raises questions as to anti-IL-10R1 mAb's role as more than just an augmentation to BCG for local bladder cancer control. Confirmatory experiments and mechanistic studies are necessary, but anti-IL-10R1 mAb shows great potential in not only local bladder cancer control, but also possibly systemic immunomodulation for the prevention of metastatic bladder cancer.

## 6. Conclusions

Bladder cancer is a disease that places significant social and financial burdens both on the patient and on society, costing nearly $4 billion annually in the U.S. BCG, which stimulates a robust immune response in most patients and has become the standard of care after surgical resection of nonmuscle invasive disease. However, despite treatment, a significant portion of patients still recur or are intolerant of BCG side effects. Multiple immunotherapies including IFN-*α*, IL-2, IL-12, and IL-10 have been investigated, either as adjuncts with BCG or as a solo replacement therapy. To date, there are a multitude of encouraging *in vitro* and murine studies; however, no clinical data has yet been reported, which is compelling enough to change the standard of care, yet many practitioners continue to use adjunctive immunotherapy based on basic science data and theoretical benefit. At our institution, for instance, BCG or BCG/IFN-*α* refractory disease is often treated with “quadruple therapy”—a combination of BCG, IFN-*α*, IL-2, and GM-CSF. The widespread use of immunotherapy for bladder cancer highlights the need for additional basic science and clinical research to further our understanding of tumor biology, human immunology, and the treatment of urothelial carcinoma.
